# Consumption Patterns of Grain-Based Foods among Children and Adolescents in Canada: Evidence from Canadian Community Health Survey-Nutrition 2015

**DOI:** 10.3390/nu11030623

**Published:** 2019-03-14

**Authors:** Syed Hamzeh Hosseini, Yanni Papanikolaou, Naorin Isalm, Patil Rashmi, Arash Shamloo, Hassan Vatanparast

**Affiliations:** 1 University of Saskatchewan, College of Pharmacy and Nutrition, Saskatoon, SK S7N 2Z4, Canada; h.hosseini@usask.ca (S.H.H.); nai695@mail.usask.ca (N.I); rashmi.patil@usask.ca (P.R.); arash.shamloo@usask.ca (A.S.); 2 Nutritional Strategies Inc, Paris, ON N3LOA3, Canada; papanikolaou.yanni@gmail.com

**Keywords:** grains, cluster analysis, consumption patterns, nutrients, socioeconomic status, fortification

## Abstract

The current analyses used data from the Canadian Community Health Survey-Nutrition 2015 to investigate grain-based food (GBF) dietary patterns of consumptions among 6,400,000 Canadian children and adolescents 2 to 18 years old. Nutrient intakes, socioeconomic differences, body mass index (BMI) *z*-scores, and intakes of several food groups were examined across the identified grain patterns of consumption. We employed *k*-mean cluster analysis to identify the consumption patterns of grain products. Based on the contributions of 21 grain food groups to the total energy intake of each individual, seven GBF consumption patterns were identified including other bread; salty snacks; pasta; rice; cakes and cookies; white bread; and mixed grains. Individuals having less than one serving of grain products were also separately categorized as no-grain consumers. Mean energy intake (kcal/day) was lowest for the “no-grain” consumers and greatest in children/adolescents consuming a “salty snacks” pattern when all GBF patterns were compared. Children and adolescents with “no-grain” and “rice” GBF consumption patterns had significantly lower intakes of several nutrients including dietary fiber, folate, magnesium, calcium, iron, zinc, thiamin, niacin, and riboflavin. No associations were observed with any of the identified GBF patterns and BMI z-scores. In addition, the socioeconomic status (SES) indicators such as household incomes and immigration status of participants were shown to be significantly different across the identified clusters.

## 1. Introduction

Grain products are one of the main staple foods across the globe. In Canada, data from the 2015 and 2004 Canadian Community Health Survey (CCHS 2.2) showed that grain-based foods (GBFs) are important sources of folate, thiamine, iron, fiber, and energy in the daily diet [1]. On average, these foods contributed 45% of folate, 41% of iron, 35% of fiber, and 25.9% of energy to the daily diets of Canadians. Canada’s Food Guide (CFG) recommends that at least half of the grain products consumed daily should be whole grains [2]. This recommendation was based on observations linking the consumption of higher servings of whole-grain to lowered risk of cardiovascular diseases and reduced body weight [3,4,5,6]. Consumption of whole grain foods is associated with lower risk of developing colorectal cancer [7], type 2 diabetes [8,9], and also cancer and mortality from all causes [10,11]. Moreover, whole grains contribute key nutrients to the diet, including dietary fiber, protein, vitamin B6, and magnesium [12].

The mandatory fortification policy in Canada has made refined grains sources of some key nutrients [13]. According to the Canadian Food Inspection Agency (2017), flour (i.e., white or refined flour) is required to be fortified with iron, folic acid, riboflavin, thiamine, and niacin [13]. Furthermore, vitamin B6, magnesium, d-3pantothenic acid, and calcium could be added to flour voluntarily [13]. However, it should be noted that only refined grains are enriched in Canada and whole wheat and whole grains are not fortified in this country [14]. Therefore, one would expect that the voluntary and mandatory enrichment and fortification of refined grains could contribute to intakes of the added nutrients closer to national recommendations. However, according to Health Canada, iron and calcium, added to refined grains, along with vitamin D and potassium are nutrients of public health concern, because a considerable portion of Canadians have below the recommended level intakes of iron, calcium, vitamin D, and potassium [15]. 

Examining the patterns of GBF consumption in the United States, Papanikolaou et al [16]. identified eight GBF patterns of consumption for U.S. children and adolescents aged 2 to 18 years. These GBF patterns were as follows: no grain; yeast bread and rolls; cakes, cookies and pies; pasta; cereals; waffles, pancakes, and French toast; salty snacks and crackers; quick bread; and finally, cooked cereals and rice. The results of that study suggested that grain consumers, in general, had a better diet quality than no-grain consumers [16]. In addition, grain consumers in the U.S. had higher intakes of some nutrients such as dietary fiber, folic acid, and iron.

A previous study conducted by our team (2017) using CCHS 2.2 data showed that both children and adults in Canada consume more refined/enriched grains than whole grain foods [17]. However, the consumption patterns of GBFs and the contributions of GBFs to the nutrient intakes of Canadians have yet to be investigated among children, adolescents, and adults. As well, the link between diverse types of grain products consumed and nutrient contributions and socioeconomic status (SES) of Canadians have not been reported based on recent Canadian nationally representative data. Therefore, the purpose of the present study was to find GBF patterns of consumption in Canadian children and adolescents aged 2 to 18 years and examine the differences in the intakes of energy, nutrients, SES, and BMI z-scores across identified clusters.

## 2. Materials and Methods

The current study used data from CCHS-Nutrition 2015, a nationally representative cross-sectional survey which includes 24,000 respondents aged one year and older living in private dwellings in Canadian provinces. Following Barr et al. (2018), we excluded children below two years of age, pregnant and breastfeeding women, and individuals with unusual energy intakes of less than 200 Kcal and more than 8000 Kcal on the interview day, resulting in the sample size of 19,797 respondents [18]. 

Along with the socioeconomic status of respondents, CCHS-Nutrition 2015 data also include dietary intake data collected via two inconsecutive 24 h dietary recalls. The second 24 h dietary intake recall was conducted on one-third of the sample for statistically estimating the usual intakes of nutrients and food items. The food consumed by each individual was classified based on several classifications including Tiers [19] and the Bureau of Nutritional Sciences (BNS) [20] classifications. Details about the dietary assessment method have been described elsewhere [21]. 

For the present analyses, we used the first 24 h dietary recall BNS classification to assess the GBF consumption patterns of children and adolescents aged 2 to 18 years. BNS classification is especially useful where the consumption of various food items is evaluated in a study. There are 53 GBFs both in the main food groups and recipe levels within the BNS classification system [20]. Based on similarities of the food groups identified, we merged them into 21 food groups. For each individual, the intake of energy from each of the 21 food groups was separately calculated on day one. Using the energy intake from each group, the contribution of that food group to the total energy intake on day one 24 h recall was employed to conduct the cluster analysis.

In this study, *k*-mean cluster analysis [22,23] was employed to identify the dietary pattern of GBFs in Canada for the age group of 2 to 18 years. Clustering has commonly been used for the identification of dietary patterns [24,25]. The *k*-means clustering is an iterative process partitioning the data into *k* groups. The procedure starts with *k* random centers where observations are assigned to the closest centroid. In the second step, the mean of observations assigned to the *k* randomly chosen centroids is calculated. Afterward, a new center is determined based on the calculated mean for each group [26]. In an iterative procedure, the second step explained above continues until the observations are grouped in specific partitions whose distances from each other are significantly maximized.

An important step in the clustering procedure is to determine the number of clusters (i.e., k*). The *k* value is initially determined by researchers based on a theoretical background. However, in the case of dietary patterns, such information does not usually exist. Therefore, k* should be determined based on a series of the methods explained below. In the first method, the kink in the scree plots of within sum of squares (WSS), the logarithm of WSS, the η^2^ coefficient (η^2^ is a measure that is very similar to the R-squared), and the proportional reduction of error (PRE) coefficient are taken into consideration when detecting k* [26]. In the second method, we used the “cluster stop” command in Stata [27] in which a measure developed by Cali$\overset{´}{n}$ski and Harabasz [28] was employed. Because the results of cluster analysis can be changed in the presence of outliers, we made an attempt to identify the outliers by use of the box plot in Stata.

Using PROC SURVEYREG in SAS 9.2 (SAS Institute, Cary, NC, USA, 2013), adjusted mean values were obtained for nutrients and energy intakes in the case of each dietary pattern identified. Covariates used to obtain adjusted mean differences include sex, age, immigration status, household income level (income decile), ethnicity (white, non-white), household education (a member of the household has a higher education certificate, none of the household members holds a higher education degree), location of residence (urban versus rural), and household food security status (the household is food secure versus the household is food insecure).

Using PROC REGRESS and PROC LOGISTIC in SAS 9.2, the differences between the SES above of individuals of each cluster were analyzed in this study as well. For income categories, we used five income levels: the lowest, low, middle, high, and the highest. The lowest income level category represents families whose income is classified as being under deciles one and two, the low-income level category contains deciles three and four and so on, where the highest income group encompasses households categorized at deciles nine and ten. It should also be noted that the deciles are adjusted for the number of household members and provinces. 

Because of the complex survey design, following the Statistics Canada guidelines, we weighted and bootstrapped the data to attain generalizable population-level results [29]. The statistical differences of nutrient intakes and SES across clusters were considered significant at the alpha level of 0.05 when no overlaps were detected between 95% confidence intervals of the estimates [30]. In our analysis, to be able to compare patterns of grain consumption with those with minimal intake of grain products, the individuals with less than one serving of GBFs were considered as “no grain” consumers.

## 3. Results

Cluster analysis of GBFs among grain consumers (i.e., ≤1 serving of grains/day) rendered seven GBF dietary consumption patterns in children and adolescents. In addition, a “no grain” consumer was distinguished from the rest of consumers. The name of each cluster was chosen based on the food group with the highest contribution in that cluster. The identified dietary patterns were “other bread,” “salty snacks,” “pasta,” “rice,” “cakes and cookies,” “white bread,” and “mixed” grains. Based on the contribution of the grain foods to energy intakes from grain (i.e., contribution within cluster), the “mixed” grains cluster was comprised as follows: whole wheat and whole grain bread, 12%; whole grain cereal, 10.1%; other bread, 9.1%; cake and cookies, 8.1%; salty snacks, 7.7%; pancakes and waffles, 7.4%; muffin, 6.9%; other cereals, 6.4%; and granola bars, 6.3%.

Our analyses represent GBF consumption of over 6,400,000 Canadians aged 2 to 18 years. On average, the 21 grain-based food groups included in the cluster analysis contributed to over one-fifth of the total energy intake of children and adolescents. Among the grain food groups considered in this study, “other bread,” “cake and cookies,” “white bread,” “pasta,” and “salty snacks” had the highest contributions to daily energy intake, respectively. The “other bread” category included several food items such as rolls, bagels, pita bread, croutons, dumplings, matzo, and tortillas according to BNS food group classification.

Table 1 presents the names and population-level percent of Canadian children and adolescents in each cluster. In addition, it shows the contribution of the top five primary GBFs of each cluster to the total energy intake and the contribution of these GBFs within each cluster. For instance, the salty snacks cluster that was identified as a dietary pattern contributes to 21.4% of daily energy intake for 6.5% of individuals 2 to 18 years old in Canada. The other four primary GBFs in this cluster are cakes and cookies, other bread, white bread, and pasta contributing to total energy intake of 2.7% to 2.3%.

### 3.1. Nutrient and Energy Intake

**Figure 1:**
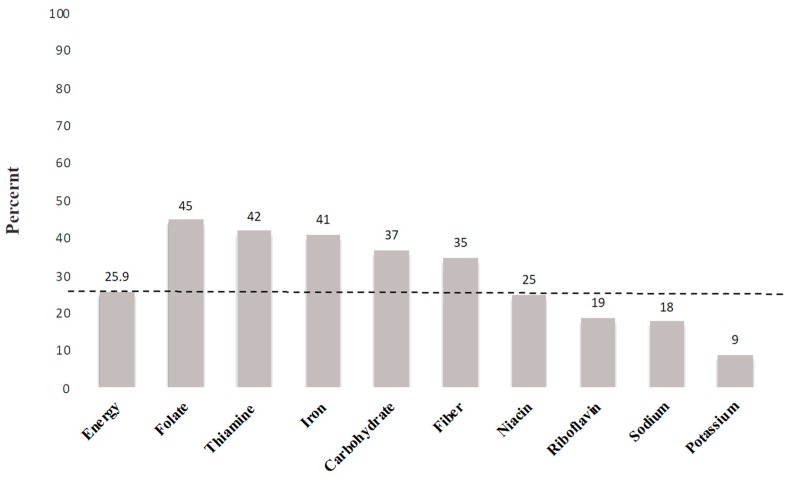
Percent sources of energy and nutrients from all grain foods in the daily diet of Canadians (≥2 years old).

Figure 1 illustrates sources of energy and nutrients of all grain foods (i.e., whole grain and enriched grain foods). While GBF is the main source of energy intake in the Canadian diet, grain foods are also important sources for dietary folate, iron, thiamin, and fiber. GBFs, on average, contributed 25.9% of daily energy intake (dashed line in Figure 1) and 37% of daily carbohydrate intake. On a daily basis, these foods provided 45% of folate, 42% of thiamine, 41% of iron, 35% of fiber, and 25% of niacin in the diet of Canadian children and adults. 

Energy and nutrient intakes in GBF identified patterns are shown in Table 2. Energy intake was lowest in children and adolescents consuming the “no-grain” cluster, while highest in children and adolescents consuming “salty snacks.” Energy intakes were not significantly different across other clusters. 

In the case of iron, calcium, and potassium as the nutrients of public health concern in Canada, and comparing the patterns of GBF consumptions, as is shown in Table 2, “no grain” consumers had significantly lower intakes of calcium and iron relative to all other grain clusters. The lowest daily calcium intakes were observed in the “no grain” and “rice” GBF patterns (654.9 mg and 741.8 mg, respectively). The greatest daily intake of calcium among clusters was observed in children and adolescents consuming the “mixed” grain pattern (i.e., 1062.5 mg/day) where whole grain and whole wheat bread, enriched grain bread, and cereals comprised approximately 30% of the cluster portfolio. We observed no statistically significant differences in the remaining patterns regarding calcium intake. Consuming a “no grains” dietary pattern was associated with significantly lower daily dietary fiber intake in comparison to all GBF patterns, with certain GBF patterns contributing greater than 6 g of dietary fiber per day versus children and adolescents avoiding grain foods (other bread: 17 ± 0.5; salty snacks: 17.5 ± 0.8; mixed grains: 16.5 ± 0.3; vs. no grains: 9.7 ± 0.5 g/day). 

The lowest intakes of iron were observed in the “no grain” and “rice” GBF patterns (6.9 mg and 10 mg, respectively). Children and adolescents consuming the mixed grain pattern had greater potassium intake (2708.5 mg/day), likely attributable to the whole and enriched grain bread and cereals in this cluster group. Vitamin D and vitamin A intakes were not significantly different among the GBF patterns for children and adolescents aged 2 to 18 years.

All GBF patterns of consumption were associated with greater intakes of iron, folic acid, riboflavin, thiamine, niacin, magnesium, and calcium in comparison with “no grain” children and adolescents. While individuals consuming a “rice” pattern had the lowest intake of several nutrients, relative to the other GBF patterns, rice consumption was associated with greater nutrient intakes in comparison to the “no grain” cluster. Children and adolescents consuming a grain pattern comprised of “pasta” had the highest intake of folic acid relative to all clusters identified. 

No significant differences were observed among several GBF dietary patterns and the “no grain” pattern for sugar intake. Consumption of a predominant “no grain” pattern was associated with significantly lower daily sodium intake in comparison to all GBF patterns, whereas a “rice” GBF pattern was associated with lower sodium intake relative to all other GBF patterns. 

### 3.2. Consumption of Other Food Groups across GBF Consumption Patterns

Table 3 illustrates the average intakes of several food groups consumed across each cluster regarding the number of servings. On average, “mixed” grain consumers had significantly higher intakes of “fruits and vegetables,” where the consumption of fruits and potato was notably higher for consumers in this group. “Mixed” grains consumers also had significantly higher intakes of “milk and alternatives” among which the individuals in this group had higher intakes of “other milk products” such as cheese and butter; this was not the case of “fluid milk” products. Among the identified consumption patterns of GBFs, “rice” consumers on average had lower intakes of “milk and alternatives” and higher intakes of “meat and alternatives” where the rice consumers tended to consume lower amounts of “processed meat” foods.

### 3.3. SES and GBF Patterns 

Table 4 shows the differences in SES and Z-BMI of the individuals across identified GBF consumption patterns along with the “no grain” group. No differences were observed across the clusters regarding BMI z-score. 

Canadian children with a dominant pattern of “rice” intake were primarily located in urban areas. Compared to the other clusters and “no grain” group, 76% of “rice” consumers were non-Caucasian, and 18% of the individual in the “rice” cluster were immigrants. Furthermore, a notable proportion of “salty snacks” consumers (67%) were the children and adolescents belonging to the households in which none of the family members had a university degree. A sizeable proportion of children (28%) in the “salty snacks” cluster were members of food insecure families, while they had higher energy intake compared to the other clusters and “no grain” group. Considering the GBF consumption pattern across five income levels, as is shown in Table 5, the percentage of individuals with “pasta,” “rice,” and “white bread” as their primary grain foods was significantly lower in the highest income level. 

## 4. Discussion 

This study is the first to identify GBF consumption patterns of Canadian children and adolescents aged 2 to 18 years. We also report the differences in the daily intake of key nutrients, other foods groups, and SES across different GBF dietary patterns. Our results indicate that the consumption of certain GBFs is better than “no grain” consumption (i.e., less than one serving per day) due to their contribution to the intake of some key nutrients. In addition, our findings imply that mandatory and voluntary fortification of GBFs has made refined grains an important dietary source of key nutrients, including iron, folic acid, riboflavin, thiamin, and niacin. The intakes of iron, folic acid, riboflavin, thiamin, and calcium were higher in specific GBF consumption patterns compared with “no grain” consumers, justifying the inclusion of these nutrients in a diet with balanced proportions of whole grains. Several GBFs such as pasta, all different kinds of bread, and cereals contributed to the higher intakes of iron and calcium, which have been identified as nutrients of public health concern in Canada by Health Canada [15]. In addition, the dietary pattern analyses of children and adolescents aged 2–18 years suggest that a high proportion of children and adolescents with dominant GBF patterns of “rice” and “salty snacks” were living in households that were immigrants, or non-Caucasian, or lower household level education, or food insecure. Moreover, the proportion of children and adolescents that were the members of families with the highest income level (deciles 9 and 10) was significantly lower in the case of “pasta,” “rice,” and “white bread” clusters. These results suggest that SES indicators play a role in the choice of GBFs in the diets of children and adolescents in Canada. 

Based on the results of our study, GBFs are important sources of some key nutrients for children and adolescents. These findings are in agreement with previous reports showing that intake from GBFs contribute to higher intakes of some shortfall nutrients in the U.S. such as iron, folate, magnesium, calcium, and fiber [16,31,32]. Studies suggest that dietary habits established early in life will be likely maintained to adulthood [33,34,35,36]. Hence, the consumption of healthy GBFs, with lower fat, sodium, and sugar content, as recommended by the current guidelines [2,37], should be encouraged among children and adolescents. Accordingly, promoting GBF consumption should be considered with caution since individuals with certain GBF patterns such as the “salty snacks” and “cake and cookies” patterns have significantly higher intakes of energy, carbohydrates, sodium, and sugar compared to other grain food patterns. 

Iron, calcium, vitamin D, and potassium are the nutrients of public health concern in Canada [15]. Our study showed children and adolescents with any pattern of GBF consumption identified have higher intakes of iron, calcium, and potassium. Hence, one could suggest that the mandatory and voluntary fortification practice of refined grains is likely successful due to the higher intake of important nutrients in enriched-grain consumers. However, among the GBFs, the “rice” consumption pattern (that is the predominant grain consumed by 10% of children and adolescents living in Canada) is linked with significantly lower intakes of iron and calcium compared to other patterns. The majority of individuals for whom rice is the primary GBFs are non-Caucasian, children and/or immigrants who live in food secure households with higher education. This finding suggests that cultural and ethnic background may play an important role in the choices of food. 

The low contribution of the “rice” pattern to folate and calcium raises concerns about the current and future health status of children and adolescents in this group. This is because the average daily intake of calcium among rice consumers is 742 mg/day, whereas the estimated average requirements (EAR) of calcium for children and adolescents under the age of 19 years is in the range of 800 to 1100 mg/day. It should also be noted that, in general, individuals with other GBF consumption patterns are likely to have average calcium intakes of below the EAR of 1100; however, rice consumers have the lowest intake of calcium after the no grain consumers. Furthermore, in the case of folate, the EAR for adolescents aged 14 to 18 years is 330 DFE (dietary folate equivalence) in mcg/day, whereas the average intake of folate for rice consumers is 304.5 DFE in mcg/day. Therefore, policies should be directed toward promoting diversified grain consumption in children and adolescents that culturally have a higher consumption of rice [38].

“Salty snacks” are the primary GBFs consumed by about 6.5% of young children and adolescents in Canada, contributing to one-fifth of their total energy intake. Our analyses showed that these individuals were more likely to live in families that were food insecure and had no members with a university degree. Our findings are in line with earlier studies implying that children in families with lower income and education levels are more likely to have unhealthy dietary choices that are linked to issues with the affordability of healthy food items [39,40]. 

Bennett’s law in economic literature implies that an increase in income is associated with a decrease in expenditures on “starchy staples” [41]. Bennett’s law is associated with the willingness to diversify one’s diet following an income increase [42]. Therefore, the lower percentage of children living in the households with the highest income level in the “pasta,” “rice,” and “white bread” groups is likely to be linked with this Bennett’s law. However, for a better understanding of the link between income and consumption of grain products along with other foods, one could investigate in the future the dietary patterns of all food groups to have a more accurate understanding of the connection between income, diet diversity, and the intake of different foods. Overall, our findings suggest that the two spectra of income may lead to food product purchases which may expose individuals to the risk of excessive intake of nutrients to limit (e.g., sodium, added sugar) and inadequate intake of other key nutrients such as folic acid.

Cluster analysis has some limitations. The cluster analysis performed in this study was based on the first 24 h dietary recall data available from CCHS 2015. The dietary intake data are self-reported and are subject to over- or under-reporting [43] that could lead to the identification of dietary patterns that do not reflect the real consumption patterns. We merged the initial 53 GBF groups from the BNS into 21 food groups. The integration of food groups was done primarily because of the similarities between items in the initial 53 groups. We also noted that the BNS classification still has some important limitations. For instance, the “other bread” group in the BNS classification includes several grain products that make it difficult to identify the dietary patterns in more detail. In our analysis, we observed that the broad category of “other bread” contributed the highest to the total energy intake of the individuals in this study. In addition, items such as English muffin is categorized into several categories including muffin and English muffin and other bread that could result in double counting of the food items identified in more than one food group. The BNS has two other food groups called “whole grain and high fiber cereals” and “other cereals” that we did not merge because the former food group includes both hot and cooked cereal as well as ready-to-eat breakfast cereals. Therefore, to account for both of these groups as well as to be able to trace the consumption of whole grain cereals versus non-whole grain cereals, these two food groups were considered as two different food groups. Nevertheless, it seems that the identified clusters in our study are distinct enough as compared to other studies conducted in the U.S. [16].

## 5. Conclusions 

Cluster analysis rendered seven distinct GBF dietary patterns for Canadian children and adolescents aged 2 to 18 years. In general, our findings suggest that grain foods can be important contributors to energy, dietary fiber, magnesium, calcium, folic acid, iron, niacin, riboflavin, thiamin, and zinc in the Canadian diet. Among the grain food consumption patterns, children and adolescents with “no grain” and “rice” consumption patterns had lower intakes of some key nutrients including, but not limited to, iron, folic acid, riboflavin, thiamine, and niacin. Our findings suggest that due to the mandatory and voluntary fortification of refined grains, these foods are important contributors to the intake of the nutrients of public health concern in Canada, especially iron, calcium, and potassium. The higher contribution to the intake of important nutrients in certain GBFs, especially those including refined grains, could justify a balanced intake of refined and whole grains. Concurrently, the present findings suggest no associations between GBF pattern consumption and BMI z-scores in Canadian children and adolescents. Therefore, Canadians can benefit from the inclusion of both enriched and whole grain food products in their diet. Furthermore, this study showed that challenging SES, such as food insecurity, lower income, and education levels as well as culture, may play an important role in the choice of grain-based foods. Targeted health promotion strategies promoting healthy food choices of grain-based foods should be designed for those at-risk populations. 

## Figures and Tables

**Table 1:** Grain consumption patterns identified from the Canadian Community Health Survey 2015 in children and adolescents (2–18 years old).

GBF Pattern	Contribution of GBFs to Total Energy Intake (%)	GBFs’ Contribution within Cluster (%)	Canadian Children in Cluster (%)	Population (*n*)
Mixed			32.9	2,100,000
Whole Grain and Whole Wheat Bread	2.9	12.0		
Whole Grain Cereals	2.5	10.2		
Other Bread	2.2	9.2		
Cakes and Cookies	2.0	8.1		
Salty Snacks	1.9	7.7		
Pancakes and Waffles	1.8	7.4		
Muffin	1.7	6.9		
Other Cereals	1.6	6.4		
Granola Bars	1.5	6.3		
Pasta	19.7	48.5	13.4	860,000
Cakes and Cookies	2.6	6.3		
Other Bread	2.3	5.6		
White Bread	2.0	5.0		
Whole Grain Cereals	2.0	4.9		
Other Cereals	2.0	4.8		
Salty Snacks	1.8	4.3		
Whole Grain and Whole Wheat Bread	1.5	3.7		
Muffin	1.5	3.6		
Granola Bars	1.4	3.3		
White Bread	17.5	51.1	11.9	760,000
Cakes and Cookies	2.7	7.9		
Other Bread	1.7	5.0		
Salty Snacks	1.7	4.9		
Pasta	1.6	4.6		
Other Cereals	1.6	4.5		
Rice	1.4	4.1		
Granola Bars	1.4	4.1		
Whole Grain Cereals	1.1	3.1		
Muffin	0.9	2.6		
Other Bread	20.6	55.0	11.8	750,000
Cakes and Cookies	2.2	5.8		
Salty Snacks	1.9	5.2		
White Bread	1.6	4.1		
Whole Grain Cereals	1.5	3.9		
Pasta	1.4	3.7		
Rice	1.3	3.5		
Granola Bars	1.3	3.4		
Other Cereals	1.2	3.3		
Whole Grain and Whole Wheat Bread	1.0	2.7		
Rice	18.8	53.5	10	640,000
White Bread	2.4	6.9		
Cakes and Cookies	2.1	5.9		
Whole Grain Cereals	1.8	5.0		
Whole Grain and Whole Wheat Bread	1.5	4.4		
Other Cereals	1.5	4.3		
Other Bread	1.4	3.9		
Salty Snacks	1.4	3.9		
Pasta	0.8	2.4		
Pancakes and Waffles	0.8	2.2		
Cakes and Cookies	21.9	51.0	10	640,000
Other Bread	3.0	6.9		
White Bread	2.6	6.1		
Pasta	2.5	5.7		
Salty Snacks	1.7	4.1		
Other Cereals	1.7	4.0		
Whole Grain and Whole Wheat Bread	1.6	3.8		
Rice	1.5	3.5		
Whole Grain Cereals	1.5	3.5		
Pancakes and Waffles	1.2	2.7		
Salty Snacks	21.4	50.0	6.5	410,000
Cakes and Cookies	2.7	6.3		
Other Bread	2.5	5.9		
White Bread	2.3	5.4		
Pasta	2.3	5.3		
Whole Grain and Whole Wheat Bread	1.7	3.9		
Granola Bars	1.6	3.7		
Other Cereals	1.5	3.4		
Pancakes and Waffles	1.4	3.2		
Muffin	1.3	3.1		

The table reports the first 10 grain food groups in each cluster ranked based on their contributions to total daily energy intakes. Whole grain cereals and other cereals were considered as two different groups because whole grain cereals include hot and cooked cereals and ready-to-eat cereals. “Whole bread” and “whole grain and whole wheat bread” were considered as two different groups. According to Health Canada (2013) [14], “whole grain bread” and “whole wheat bread” are two different products, therefore for the sake of the objectivity of the analysis, we did not merge these two groups, although the food group of “whole grain and whole wheat bread” includes both “whole grain bread” and “whole wheat bread”.

**Table 2:** Adjusted mean (SEM) of daily nutrients and energy intakes for all clusters of grain-based foods among children and adolescents aged 2–18 years.

	No Grain	Other Bread	Salty Snacks	Pasta	Rice	Cakes and Cookies	White Bread	Mixed	*p*-Value
Energy Intake	1259.7 * (63.4)	1848.3 (44)	2060.3 *† (79)	1757.1 (37.5)	1647.3 (53.3)	1881.1 (50.5)	1743.4 (39.1)	1939.8 (31.4)	0.99
% Energy from Carbohydrates	48.5 * (1.4)	55 (0.5)	54.8 (0.6)	54.6 (0.5)	54.3 (1)	54.3 (0.5)	54.5 (0.5)	51.8 *† (0.3)	<0.001
Calcium (mg)	654.9 * (52)	932.6 (32.6)	953.8 (42.2)	962.2 (27.5)	741.8 *† (34.2)	963.1 (31.8)	880.9 (28)	1062.5 *† (21.3)	<0.001
Total Carbohydrates (g)	154.9 * (9.3)	253.9 (5.7)	280.6 * (9.8)	242.6 (5.9)	221.5 (8.2)	258.7 (7.2)	237.4 (5.1)	251 (4.1)	<0.001
Folate (DFE in mcg)	204.8 * (11.9)	498.7 *† (16.9)	400.6 (18)	556.3 *† (15.9)	304.5 *† (15.5)	422.8 (15.7)	462.4 (13.1)	418.2 (9.8)	<0.001
Fat (g)	50.8 (3)	62.6 (2)	76.7 *† (3.9)	58.7 (1.4)	52.6 † (2.4)	68.6 (2.2)	59.8 (1.8)	71.8 (1.5)	<0.001
Dietary Fibers (g)	9.7 * (0.5)	17 (0.5)	17.5 (0.8)	14.9 (0.4)	12.9 † (0.7)	14.3 (0.4)	13.9 (0.3)	16.5 (0.3)	<0.001
Folic Acid (mcg)	39.5 * (4.8)	160.9 (6.6)	128.1 (7.4)	213.4 *† (7.6)	68.3 *† (5.3)	130.8 (5.2)	155.8 (4.6)	111.2 *† (3.8)	<0.001
Iron (mg)	6.9 * (0.4)	13.2 (0.4)	12.1 (0.5)	12.4 (0.3)	10 *† (0.4)	12.8 (0.4)	12.3 (0.3)	12.5 (0.3)	<0.001
Magnesium (mg)	176.5 * (9.3)	261.4 (7.8)	274.5 (11)	254.9 (7)	245.1 (9.4)	243.5 (6.8)	239.3 (5.8)	281.7 (5)	0.05
Niacin (mg)	22.9 * (1.7)	34.9 (1)	32.7 (1.4)	33.8 (0.9)	33.6 (1.5)	30 (0.8)	32.8 (1)	36.2 (0.8)	<0.001
Potassium (mg)	2012.1 (123.5)	2269.5 (56)	2402.4 (83.9)	2327.9 (60.4)	2249.6 (69.2)	2232.2 (60.9)	2185.7 (50.1)	2708.5 †* (45.7)	<0.001
Vitamin A (mcg)	508.6 (43.3)	545.7 (22.7)	534.2 (29.5)	618.6 (26.4)	515.4 (26)	633.8 (30.3)	613.4 (61.8)	674.8 (19.6)	<0.001
Riboflavin (mg)	1.3 * (0.1)	1.8 (0.1)	1.8 (0.1)	1.9 (0.1)	1.6 *† (0.1)	1.8 (0.1)	1.9 (0.1)	2 (0)	<0.001
Sodium (mg)	1680.1 * (115)	2654.4 (75.2)	2988.7 *† (121.6)	2474 (58.5)	2190.8 *† (81.1)	2475.3 (80.2)	2689.6 (78.4)	2681 (51.9)	<0.001
Sugars (g)	88.7 (7.2)	100.7 (2.7)	115.2 (5.1)	97.9 (3.1)	79.1 † (3.4)	128.9 † (4.5)	105 (2.7)	120.7 (2.5)	<0.001
Thiamin (mg)	0.9 * (0.1)	1.8 (0.1)	1.5 (0.1)	1.8 (0.01)	1.3 *† (0.1)	1.5 (0.03)	1.6 (0.01)	1.6 (0.02)	<0.001
Zinc (mg)	7 * (0.7)	9.2 (0.3)	9.3 (0.4)	8.8 (0.2)	10 (0.5)	8.5 (0.3)	8.5 (0.3)	10.2 (0.2)	<0.001

* Significantly different as compared to all GBF patterns (*p* < 0.05). † Significantly different as compared to other clusters (*p* < 0.05). DFE: dietary folate equivalence.

**Table 3:** Average intakes of food groups consumed across identified clusters (number of servings).

	No Grain	Other Bread	Salty Snacks	Pasta	Rice	Cakes and Cookies	White Bread	Mixed
**Total Fruits and Vegetables**	**4.3** **(0.37)**	**3.8** **(0.15)**	**4.2** **(0.26)**	**4** **(0.2)**	**3.6** **(0.2)**	**3.8** **(0.15)**	**3.8** **(0.14)**	**4.9 †** **(0.13)**
Fruits (excluding fruit juice)	1.2 (0.13)	1.2 (0.1)	1.4 (0.19)	1.4 (0.14)	1.2 (0.11)	1.3 (0.08)	1.2 (0.07)	1.7 (0.07)
Dark Green Vegetables	0.3 (0.04)	0.3 (0.03)	0.2 (0.04)	0.3 (0.04)	0.4 (0.04)	0.3 (0.04)	0.2 (0.03)	0.3 (0.03)
Orange Vegetables	0.2 (0.04)	0.1 (0.02)	0.1 (0.02)	0.1 (0.01)	0.1 (0.02)	0.2 (0.03)	0.1 (0.01)	0.2 (0.02)
Potato	0.6 (0.09)	0.4 (0.05)	0.4 (0.06)	0.3 (0.03)	0.4 (0.07)	0.4 (0.05)	0.4 (0.04)	0.6 † (0.04)
Other Vegetables	1 (0.15)	0.8 (0.06)	0.9 (0.1)	1.1 (0.07)	0.7 (0.07)	0.8 (0.08)	0.8 (0.06)	0.9 (0.04)
**Total Milk and Alternatives**	**1.6** **(0.21)**	**1.9** **(0.11)**	**2.1** **(0.15)**	**2.2** **(0.09)**	**1.8 †** **(0.11)**	**2.2** **(0.1)**	**2** **(0.09)**	2.5 *† **(0.07)**
Fluid Milk or Soy Milk	0.9 (0.1)	1 (0.07)	1 (0.11)	1.2 (0.07)	1 (0.08)	1.2 (0.07)	1.1 (0.06)	1.3 (0.04)
Other Milk Product	0.7 (0.16)	1 (0.07)	1.1 (0.09)	1 (0.07)	0.7 (0.08)	1 (0.07)	1 (0.06)	1.2 *† (0.05)
**Total Meat and Alternatives**	**1.3** **(0.14)**	**1.4** **(0.08)**	**1.3** **(0.1)**	**1.2** **(0.06)**	1.9 *† **(0.14)**	**1.2** **(0.07)**	**1.5** **(0.08)**	**1.7** **(0.07)**
Poultry	0.5 (0.11)	0.5 (0.05)	0.4 (0.06)	0.3 (0.04)	0.7 (0.08)	0.3 (0.04)	0.3 (0.04)	0.6 (0.05)
Beef	0.2 (0.05)	0.3 (0.03)	0.3 (0.05)	0.2 (0.02)	0.4 (0.08)	0.2 (0.02)	0.3 (0.04)	0.3 (0.03)
Legumes	0.1 (0.03)	0.2 (0.03)	0.2 (0.04)	0.1 (0.02)	0.2 (0.04)	0.1 (0.02)	0.3 (0.05)	0.2 (0.03)
Eggs	0.1 (0.04)	0.2 (0.03)	0.1 (0.02)	0.1 (0.01)	0.2 (0.02)	0.1 (0.01)	0.2 (0.02)	0.2 (0.01)
Processed Meat	0.3 (0.07)	0.3 (0.03)	0.3 (0.05)	0.2 (0.03)	0.1 *† (0.02)	0.4 (0.06)	0.4 (0.03)	0.3 (0.02)

* Significantly different as compared to all GBF patterns (*p* < 0.05). † Significantly different as compared to other clusters (*p* < 0.05). The rows with the bold fonts are the primary food groups and the rows with regular font are the sub groups.

**Table 4:** Socioeconomic difference across identified clusters of grain-based foods. Age group: 2–18 years.

	No Grain Consumers	Other Bread	Salty Snacks	Pasta	Rice	Cakes and Cookies	White Bread	Mixed
Mean age (SEM)	9.94 (0.5)	11.29 (0.26)	9.4 (0.42)	9.7 (0.31)	10.4 (0.38)	9.12 (0.26)	9.8 (0.25)	9.7 (0.15)
Male (%)	50	48	52	42 †	50	49	50	54
Caucasian (%)	64	72	72	67	24 *	73	70	76
Education (% university grad) ^1^	43	41	33 †	45	49	48	38	47
Food secure (%)	81	84	72 †	86	85	87	84	85
Urban (%)	76	81	79	86 †	93 †	78	82	80
Immigrant (%)	8	8	7	8	18 *	6	10	8
Mean Z_BMI (SEM) ^2^	0.9 (0.2)	0.5 (0.1)	0.8 (0.2)	0.4 (0.1)	0.3 (0.1)	0.4 (0.2)	0.6 (0.1)	0.4 (0.1)

* Significantly different as compared to all GBF patterns (*p* < 0.05). † Significantly different as compared to other identified clusters (*p* < 0.05). All data are weighted and bootstrapped to represent population-level information. ^1^ For children, these data reflect whether a member of the household is or is not a university graduate. ^2^ For those aged 2–18 years, based on BMI *z*-score for age and sex.

**Table 5:** The percentage of Canadian children and adolescents in five income levels for each dietary pattern.

	Income Level				
	The Lowest (%)	Low (%)	Middle (%)	High (%)	The Highest (%)
No Grain Consumers	25.17	25.55	21.2	17.9	10.18
Other Bread	17.02	22.72	22.68	19.34	18.23
Salty Snacks	25.21	16.62	25.25	17.67	15.24
Pasta	21.29	21.81	21.88	22.19	12.83 *
Rice	29.41	29.41	16.6	16.86	7.72 *
Cake and Cookies	19.81	12.75 *	29.4 *	20.84	17.23
White Bread	30.21	18.7	23.39	18.5	9.21 *
Mixed	19.79	18.22	23.55	21.65	16.79

* Significantly different as compared to other income levels (*p* < 0.05).
